# PARQ: A Complexity-Consensus Aware Automatic Assessment of Motor Disease Severity in Parkinson’s Disease

**DOI:** 10.21203/rs.3.rs-8694861/v1

**Published:** 2026-02-09

**Authors:** Isha Chakraborty, Kaitlyn Trushenski, Salman Siddique Khan, Yutong Taneff, Arjun Tarakad, Charenya Anandan, Steven Thomas Bellows, Abdullah Yasir Yilmaz, Guha Balakrishnan, Nora Vanegas-Arroyave, Ashutosh Sabharwal

**Affiliations:** 1 Electrical and Computer Engineering, Rice University, 6100 Main St, Houston, 77005, Texas, U.S.A.; 2 Department of Neurology, Baylor College of Medicine, 7200 Cambridge St., Houston, 77030, Texas, U.S.A.

**Keywords:** Parkinson’s Disease, inter-rater variability, automated video-based assessment

## Abstract

Parkinson’s Disease (PD) is the fastest growing neurodegenerative disease, creating an urgent need for scalable objective clinical approaches to assess and monitor cardinal PD features (bradykinesia, rigidity, tremor, and gait dysfunction). Currently, the evaluation of PD features relies on expert assessments using the Movement Disorders Society - Unified Parkinson’s Disease Rating Scale part III (MDS-UPDRS-III), which provides a standardized framework for quantifying motor disease severity. However, this approach relies on the availability and expertise of trained clinicians, limiting scalability and introducing variability related to the experience of the raters and task-specific complexity. Inter-rater variability arises from a fundamental consensus-complexity tradeoff inherent to visually rated motor tasks: tasks with less visual cues tend to elicit lower inter-rater variability (greater rater agreement) whereas tasks with more visual cues are associated with greater disagreement. To address these challenges, we introduce PARQ, a deep learning based platform to automatically quantify PD motor disease severity from routine clinical videos. Rather than predicting a single severity score, PARQ estimates both the expected motor severity and the underlying distribution of expert ratings, enabling task-specific, consensus-aware motor severity prediction. We evaluate PARQ on a clinical dataset of 48 patients across 8 tasks with three independent expert ratings per video. PARQ achieves 90% accuracy on high-consensus tasks and 80% on low-consensus tasks, demonstrating robustness to systematic rater disagreement. PARQ delivers task-specific, distribution-aware severity estimates across most visually rated MDS-UPDRS-III tasks, offering a foundation for objective PD motor disease severity assessment.

## 1 Introduction

Parkinson’s Disease (PD) is a chronic neurodegenerative condition characterized by a combination of motor and non-motor symptoms. PD is also the fastest-growing neurological condition worldwide, with prevalence projected to double next decade as populations age [1, 2]. Despite substantial progress in the development of PD biomarkers, the diagnosis and longitudinal monitoring remain clinically grounded on the evaluation of cardinal motor symptoms, including tremor, rigidity, bradykinesia, and gait dysfunction.

In routine clinical practice, the Movement Disorder Society - Unified Parkinson’s Disease Rating Scale Part III (MDS-UPDRS-III) is the most widely used instrument to assess the motor symptoms of PD [3, 4]. The MDS-UPDRS-III consists of a structured assessment in which clinicians rate patient performance on a series of motor tasks using a 5-point ordinal scale. MDS-UPDRS-III ratings are used to track disease progression, monitor treatment progress [5], support eligibility for advanced treatments such as Deep Brain Stimulation [6], and serve as outcome measures in clinical trials [7, 8]. However, clinical ratings are inherently subjective, varying with clinical experience [9, 10], longitudinal care [11, 12], and subtle interpretation of motor cues, leading to substantial inter-rater variability [13–15]. In addition, the reliance on human raters limits the accessibility of state-of-the-art PD care to specialized centers, which remain concentrated in metropolitan areas [11, 16, 17]. Such barriers have motivated the development of recording-based motor assessment systems [18–23], particularly video-based and wearable technology, to examine movement tasks. Most existing video-based systems, however, focus on narrow subsets of tasks and thereby lack the capacity to generalize across the broad range of MDS-UPDRS-III components [4]. Importantly, as highlighted in a recent review by Martinez-Garcia-Pena et al [24], prior work has largely treated expert ratings as a single ground truth, without explicitly modeling inter-rater disagreement and variation across tasks of increasing visual complexity.

**Fig. 1:**
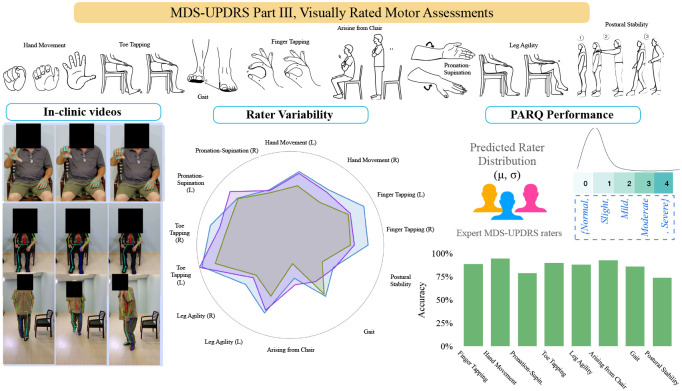
PARQ overview. The MDS-UPDRS-III assessments comprises of multiple visually rated motor tasks as shown in the top of the figure which PARQ analyzes uses in-clinic video recordings (left). Expert ratings for each task exhibit substantial inter-rater variability (center), motivating PARQ’s distribution-aware prediction of both the mean and variance of rater scores (right). PARQ integrates clinical domain knowledge with data-driven features derived from motion to predict severity estimates aligned with expert raters.

To address these gaps, we sought to develop PARQ, a platform for probabilistic, end-to-end deep learning grounded in clinical knowledge and kinematic behavior for automated assessment of bradykinesia and gait components of the MDS-UPDRS-III, captured using a standard consumer-grade mobile device as seen in Figure 1. With PARQ, we envision a new paradigm for PD assessment in which a patient may go to their local center equipped with a well-lit room and human attendant, and record their performance of the tests with an iPad-like camera device. The attendant must only ensure that all tests are performed correctly, but is not responsible for rating the patient’s performance.

Achieving a comprehensive, uncertainty-aware approach to automating the rating of MDS-UPDRS-III required rethinking both the learning objectives and modeling strategy. Compared to existing platforms, the key innovations of PARQ are *what it learns* and *how it learns*. Using a clinically realistic dataset from PD patients obtained at the Baylor College of Medicine, PARQ explicitly models the tradeoff between task complexity and rater consensus. First, motivated by the tradeoff between rater agreement (how consistently raters score a task) and task complexity (how difficult a task is to rate based on the number of visual cues), PARQ predicts not only the expected severity score but also the expected level of expert consensus by modeling the distribution of ratings (through the mean and standard deviation). Second, PARQ combines clinically grounded features directly from the MDS-UPDRS guidelines with data-driven temporal features through a deep learning architecture and a distribution-aware loss function. Our hybrid design helps PARQ predict with expert variability in mind.

By moving beyond non-deterministic scoring towards clinically realistic assessments, PARQ offers a robust and objective system for real-world PD motor severity evaluations. As PARQ operates on standard clinical videos, it can be easily integrated into telemedicine workflows, clinics, or remote clinical trials, enabling frequent, care-driven monitoring. PARQ lays the groundwork for data-driven neurological care that reflects the inherent complexity and subjectivity of expert clinical assessment.

## 2 Results

### Complexity-consensus tradeoff

**Fig. 2:**
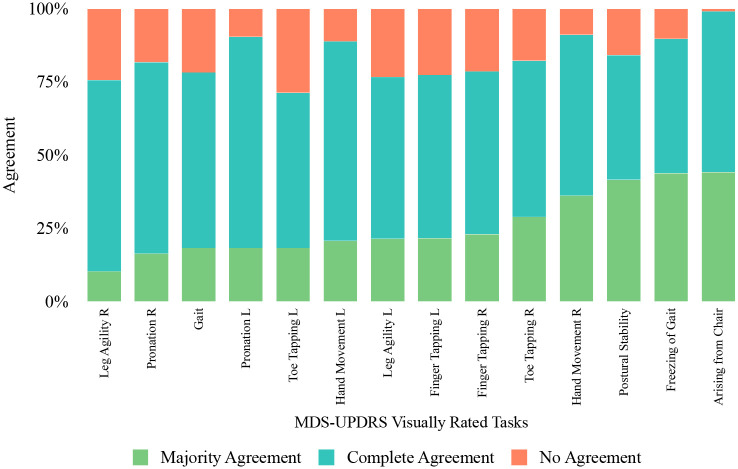
Inter-rater variability among 3 expert raters across patients and tasks. The probability of complete agreement (three raters) is below 50% on average. Majority agreement (two or more raters) is higher, approximately 70–75%

We first present results of inter-rater agreement across three expert raters and 8 motor tasks in our dataset in Figure 2. We labeled ratings in which all raters are in agreement as *complete agreement* and those in which two out of three raters are in agreement as *majority agreement*. We calculated the *agreement percentage* as the number of ratings that are agreed on for both complete and majority agreement divided by the total number of ratings to yield a complete agreement, majority agreement, and no agreement percentage. Figure 2 demonstrates substantial variability across raters, with complete agreement occurring in fewer than 50% of ratings and majority agreement in 70–75%.

**Fig. 3:**
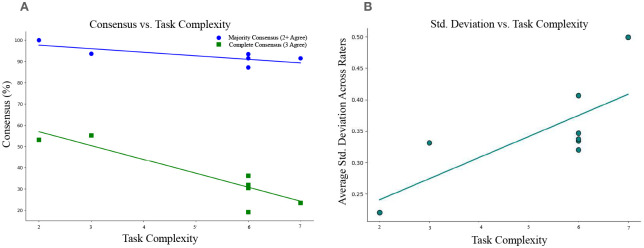
Rating complexity inversely correlates with rater agreement and positively correlates with standard deviation. Each point represents a single MDS-UPDRS Part III task (left/right collapsed). 3A. Representation of agreement across tasks (blue = “majority” or 2 rater agreement, green = “full” or 3 rater agreement) in relation to task complexity (X axis). Both full and majority agreement across three expert raters declines as the number of behavioral subcomponents (task complexity) increases, highlighting the visual ambiguity of complex or overlapping movements. 3B. Positive linear relationship between the average standard deviation of rater scores across patients and task complexity.

We further examined the effect of *task complexity* on inter-rater variability. We measured task complexity by the number of behavioral cues that raters must monitor simultaneously evaluate to assign a score taken directly from the MDS–UPDRS Part III guidelines (see Supplementary Table 3). For example, the Arising from chair task require evaluation of two cues (use of arms, rising speed), whereas the Gait task requires the evaluation of seven cues (stride amplitude, stride speed, foot lift, heel strike, turning, arm swing, use of assistive device). Figure 3 shows that agreement declines monotonically with task complexity. Linear regression models fit to these data yield slopes of −6.7 (*r*^2^ = 0.86, *p* < 0.001) for complete agreement and −1.67 (*r*^2^ = 0.51, *p* = 0.045) for majority agreement (for rater-pair agreement comparisons, see Supplementary Figure 1). The average standard deviation of rater scores also increased with task complexity (slope = 0.03, *r*^2^ = 0.55, *p* = 0.035).

Together, these results establish a quantitative *complexity–consensus tradeoff* in visually rated MDS-UPDRS-III tasks with those requiring integration of a greater number of behavioral cues (complexity) being associated with lower inter-rater agreement(consensus). The complexity-consensus tradeoff supports the need for models and metrics that account for task-specific variability in expert judgment.

### Distribution-aware prediction of expert consensus

Next, we evaluated PARQ performance on modeling the distribution of rater scores per task. As motivated above, we propose to learn both mean and standard deviation of ratings. Conventional “accuracy-within-one ±1” class accuracy, commonly used in PD literature[18, 25], inherently makes an assumption that all tasks have equal complexity and thereby ignores the distribution of consensus levels across tasks. For high-consensus tasks (e.g., Arising from Chair), ±1 is overly permissive, while for low-consensus tasks (e.g., Gait) it is overly strict. Thus, we also propose a *distribution-aware accuracy*: a prediction is correct if its predicted mean $\hat{\mu }$ lies within one empirical standard deviation (*σ*) of the rater distribution for that specific task. Our proposed criterion tightens for high-consensus tasks and relaxes for tasks with broader rater distributions, aligning performance assessment with task-dependent rater uncertainty. For example, if raters assign scores of 1, 2, and 2 (mean = 1.67, SD = 0.58), predictions between 1.09 and 2.25 are considered correct, as opposed to the wider interval of considering scores 0–3 correct under the fixed ±1 rule.

With both the ±1 metric and our proposed *distribution-aware* metric, PARQ achieved high alignment with expert ratings across all tasks with all results being shown in Table 1 (see Section 3 for details on our Methods). Accuracy exceeded 90% for high-consensus tasks such as Leg Agility, Arising from chair, and Postural stability, and remained 75–85% for lower consensus tasks (e.g., Toe Tapping, Hand Movements) under ±1 metrics and 50% (within 1 std. deviation) and 67% (within 1.5 std. deviation) on average under our distribution-aware metrics (see Supplementary Figure 4 for expanded results on individual MDS-UPDRS-III tasks).

### Predictability of MDS-UPDRS-III composite scores across medication states

**Fig. 4:**
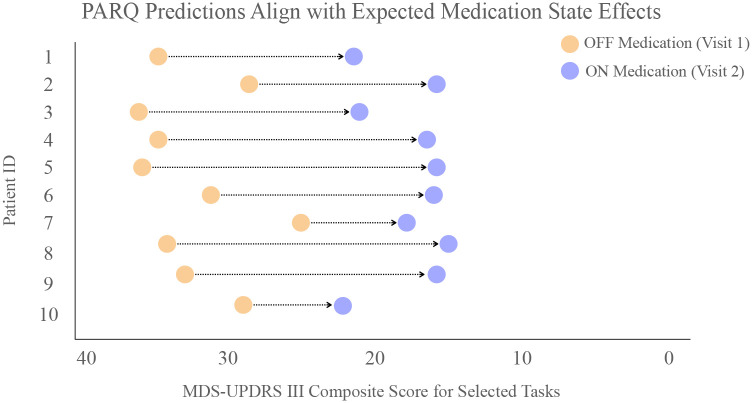
Predicted total motor severity scores for each patient in the OFF- to ON-medication condition. Arrows point from the OFF-medication prediction (blue) to the ON-medication prediction (green). For all patients, the predicted severity decreases in the ON medication state, reflecting the expected clinical improvement and demonstrating that the model is sensitive to capture the typical therapeutic effect of dopaminergic treatment.

We next evaluated model behavior during ‘ON’ and ‘OFF’ dopaminergic medication conditions. Across all tasks, mean accuracy differed by less than 5% between medication states, yet residual distributions broadened during ‘off’ examination (Table 2). Table 2 further summarizes task-specific prediction accuracy, stratified by medication state. For example, Pronation-Supination demonstrates higher accuracy in the OFF state than the ON state, whereas tasks such as Toe Tapping shows little difference across states. These variations likely reflect changes in motor severity which is typically higher OFF medication and reduced ON medication, when treatment stabilizes motor output. As expected, PARQ prediction for ON medication patients generally trend downwards as compared to OFF medication as the medication improves evaluated motor severity (as shown in Figure 4).

**Fig. 5:**
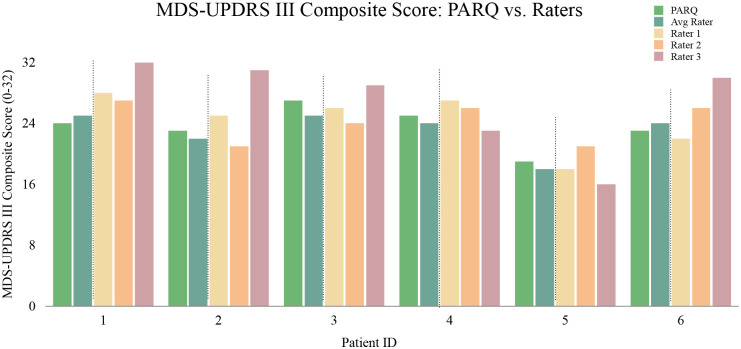
Comparison of predicted, average, and individual composite motor scores across patients Bars represent the predicted, averaged, and individual composite MDS-UPDRS-III scores for a subset of individuals. The predicted composite score generally tracks closely with the average of the three human raters. Higher divergence is associated with larger variability across raters, illustrating that disagreement between raters naturally limits how closely any model can match the average rater on every patient.

Since the clinical interpretation of motor impairment depends on the composite MDS-UPDRS-III score, evaluating how our task specific predictions aggregate into a total composite score and how our PARQ-derived composite compares with rater composite scores is a critical validation step. Figure 5 compares the predicted PARQ MDS-UPDRS-III composite score with the individual and averaged rater scores for a subset of patients. PARQ’s predictions closely follow the mean of the three raters across the cohort, even in cases with substantial inter-rater disagreement. We observe a structural limit in evaluation: when inter-rater variability is high, no single model can simultaneously agree with all raters. Nevertheless, the tight alignment between the predicted and average composite score suggests we effectively capture rater consensus on overall motor disease severity.

### Hybrid feature learning yields sample-efficient and robust performance

To model central tendency and variance under limited labeled data, we implemented a a hybrid Convolutional Neural Network – Multilayer Perceptron (CNN–MLP) architecture. The CNN branch learns spatiotemporal motion cues from pose trajectories, while the MLP branch encodes clinically derived kinematic descriptors defined by the MDS-UPDRS-III guidelines (e.g., tap frequency, amplitude, rhythm, and stride). The dual-branch design has the potential to capture both low-level dynamics and high-level clinically relevant information (discussed further in the Methods 3).

**Fig. 6:**
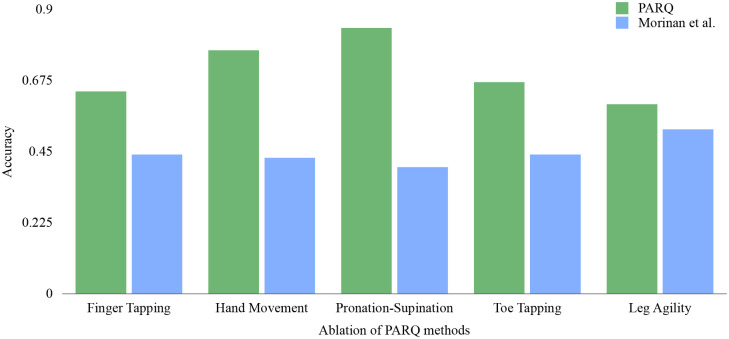
Comparison of classification accuracy of PARQ vs. Morinan et al. [18] For each of the five tasks addressed by Morinan et al., PARQ was adapted to perform binary classification to match the evaluation protocol of Morinan et al. We report accuracy for each task with PARQ consistently outperforming the competitive baseline.

Ablation experiments (see Supplementary Table 4) confirm the benefit of the dual-branch architecture. The full hybrid CNN–MLP achieved the highest distribution-aware accuracy, demonstrating superior sample efficiency and robustness, and outperformed the previously published methods [18] under matched evaluation in Figure 6.

## 3 Methods

### Automatic Task Duration Extraction from Raw Videos

Raw video recordings in our dataset include relevant patient motor task executions and irrelevant non-task actions such as post-assessment interactions. Therefore, we first developed an automated signal extraction pipeline based on identifying the relevant segment of each video corresponding to task execution. We used OpenPose [26], MediaPipe [27], and RTMPose [28] to extract the relevant joint keypoints from each frame of each video with a minimum confidence threshold of 0.5, and temporally smoothed each joint’s trajectory with a moving average filter. PARQ supports the use of any of the above pose estimation frameworks. We report empirical results using RTMPose [28] for consistency. We automatically detected task execution onset by computing the velocity and displacements of all joint trajectories individually and identifying peaks exceeding task-specific thresholds derived from training data. We detected the end of a task when joint trajectories return to baseline velocity and displacement values. For tasks performed bilaterally (e.g., evaluated on both left vs. right limb), we determined the active side by detecting which limb exhibited dominant movement in the identified window. We then trimmed videos to task-focused clips using these detected task onset and ending frames.

### Task-Specific Signal and Feature Extraction

**Fig. 7:**
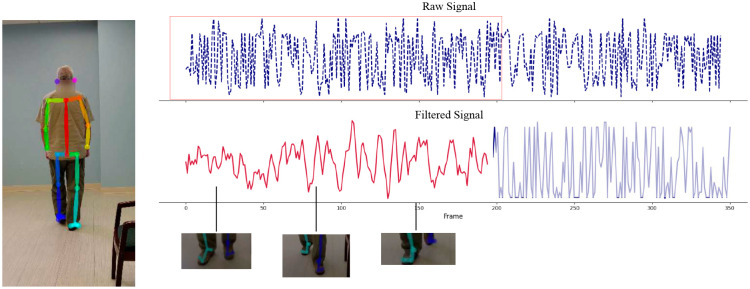
Snapshot of pose estimation components from an in-clinic video using OpenPose [26] for visualization. The raw signal is the unfiltered kinematic output derived from joints of interest from pose detection models. To identify the region of interest, we apply signal processing techniques like peak detection and Savitzky–Golay smoothing. The pink dotted signal designates the extracted region of interest. Selected frames of the feet are shown below.

To focus model inference on clinically relevant kinematics, we constructed a set of input features from task-specific motion signals guided by the MDS-UPDRS-III protocol with an example snapshot of a task shown in Figure 7. First, for each task, we isolated a subset of joints relevant for clinical scoring, and constructed task-specific signals from these joint trajectories (see Table 3). For example, finger tapping relies on the thumb and index finger movement, pronation–supination on wrist rotation, and leg agility on vertical knee displacement. Our targeted approach minimizes the inclusion of movements irrelevant to each task (e.g., trunk motion during finger tapping), which can introduce noise.

We further designed and extracted simple features from each signal for each task summarized in Table 3. We derived up to 14 kinematic features from each task informed by the MDS-UPDRS-III scoring guidelines and feedback from expert raters. MDS-UPDRS-III emphasizes motion characteristics such as speed, amplitude, rhythm, smoothness, and the slowing of movement across a repetitive motion. Accordingly, we computed a set of features aligned with these concepts: average velocity, acceleration, rate of change of acceleration, period between motion cycles, and peak-to-trough displacement amplitude.

### Model Architecture and Loss Function

**Fig. 8:**
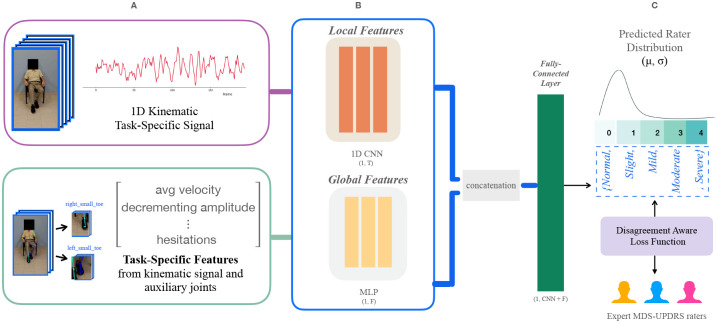
PARQ model overview. Patient videos (A) collected with the Kelvin PD Platform on an iPad during a routine assessment of the MDS-UPDRS part III exam. We extract kinematic coordinates from the human pose skeleton to model both micro and macro movement. The CNN (A, B) takes an input of the task-specific kinematic signal. The input to the MLP (A, B) is the task specific features from the 1D kinematic signal and auxiliary joints when relevant. We use the concatenation of the CNN and MLP features to predict a final severity class distribution (C), modeling a group of expert raters with our custom Disagreement Aware Loss function.

We sought to learn rich representations in a data-driven manner, while also retaining clinically interpretable features. To this end, we developed PARQ with a dual-branch architecture, shown in Figure 8. The fused representation of the CNN and the MLP allows the model to potentially leverage both data-driven pattern discovery and established clinical insight. The first branch is a convolutional neural network (CNN) that directly processes the task-specific signal waveform as a temporal sequence using a sequence of 1D convolutions with batch normalization, ReLU activations, and max-pooling operations. Typically, a CNN can capture dynamic patterns in movement (e.g. rhythm, velocity, and amplitude decay) without imposing any predefined assumptions about which temporal features are relevant [29]. However, clinical scoring is not based solely on just kinematics; it also reflects expert interpretation of specific movement qualities, often summarized by visually observed cues (e.g., smoothness, slowness, and amplitude). To explicitly embed domain knowledge, the second branch is a multilayer perceptron (MLP) that receives up to 14 task-specific features derived from clinical guidelines and expert feedback (detailed later in the Discussion 3). The feature embeddings from both branches are concatenated and passed through a final layer that outputs *K*−1 logits used in the CORAL ordinal regression framework [30]. The expanded architecture is visualized in Supplementary Figure 2.

#### Fine-Grained Ordinal Prediction

MDS-UPDRS ratings are ordinal (0 = normal to 4 = severe), where the difference between adjacent scores is ordered but not necessarily linear. Treating the scores as categorical (via softmax) would ignore the structure, while treating them as continuous (via regression) would ignore their discrete nature. To address the limitations of choosing to represent the scoring as either categorical or continuous, we use the CORAL (Cumulative Ordinal Regression for Neural Networks) framework [30], which outputs *K*−1 cumulative logits representing the probability that a sample’s severity is greater than each threshold. Our approach naturally enforces ordinal structure of the ratings and yields a probability distribution across severity levels, from which we compute the expected severity score (mean) and prediction variance.

#### Disagreement Aware Loss Function

Given the rater variability present in both our dataset and in reported in the literature[12–15], models must capture both central tendency and rating variability rather than assuming a single “ground-truth” label. We addressed this by designing a disagreement-aware loss that jointly estimates the mean severity score and uncertainty:

(1) $${ℒ}_{\text{total}}=\underset{\text{Prediction}\;\text{Accuracy}}{\underset{︸}{{ℒ}_{\text{Huber}}\left(\mu_{i},t_{i}\right)}}+\lambda \cdot \underset{\text{Uncertainty}\;\text{Regularization}}{\underset{︸}{\sigma_{i}^{2}}}$$

Here, ${ℒ}_{\text{Huber}}$ penalizes deviation from the ground truth mean rating *t*_*i*_, while the regularization term discourages unwarranted uncertainty in confident predictions. The weighting term *λ* is tuned via cross-validation.

When raters exhibit complete agreement, the model is encouraged to produce confident predictions (low $\sigma_{i}^{2}$), whereas disagreement permits higher predicted variance, reflecting the ambiguity inherent to clinical judgment. Our formulation aligns the model with expert practice and improves interpretability by distinguishing between confident, consensus-driven estimates and predictions where expert ratings themselves are variable.

#### Training and Validation

We trained a separate PARQ model for each of the nine MDS-UPDRS Part III motor tasks. Each model receives as input the task-specific signals described in Sections of the Methods 3 and 3. All models were trained for 100 epochs using Adam optimizer with a learning rate of 1 × 10^−3^ and batch size of 32. Each batch consists of CNN inputs reshaped to (batch, 1*, T*) and handcrafted features as (batch, number of features).

### Dataset Overview

We use clinical video recordings of standardized MDS-UPDRS-III motor tasks as input to PARQ as shown in Figure 8. The dataset included videos from 48 patients (26 Male, 22 Female, Mean Age of 66) for 8 MDS-UPDRS-III tasks who met established clinical diagnosis criteria for PD and were being considered for deep brain stimulation surgery [31], more details about the dataset can be found in Supplementary Tables 1 and 2. Thus, videos are representative of a cohort of individuals with moderate PD, complicated by motor fluctuations or medication refractory tumor [32, 33]. The study was conducted in accordance with institutional and national ethical standards and was approved by the Baylor College of Medicine Institutional Review Board (protocol H-53665). All participants provided written informed consent for the use of de-identified videos prior to data collection. Standardized MDS-UPDRS-III motor tasks were recorded in the ‘OFF’ and ‘ON’ dopaminergic medication state during clinic visits at the Baylor College of Medicine. All videos were independently rated by three expert raters (N.V., Y.T., and Y.Y.), yielding 1,344 videos (25,920 seconds of video) and 4,032 ratings. By explicitly capturing the inherent inter-rater variability of clinical MDS-UPDRS ratings, our dataset provides a realistic representation of PD assessments. The alignment with practical rating variability constitutes a key foundation and clinical contribution of PARQ.

## 4 Discussion

The assessment of PD features using automated video-based assessments has advanced rapidly, however despite well-documented inter-rater variability in clinical scoring, existing systems assume a single ground-truth label per motor task. We introduce PARQ, an automated distribution-aware platform for quantifying motor severity of PD from clinical video data. Across 1,344 rated patient videos spanning 8 MDS-UPDRS-III tasks, we show that expert disagreement increases with task complexity, revealing a reproducible complexity–consensus tradeoff. Leveraging this complexity–consensus as a quantifiable feature, PARQ accurately predicts both mean severity and rating variability, which remains robust across medication states and disease severities, achieving strong generalization through hybrid feature learning.

A critical insight of our work is that inter-rater agreement is strongly influenced by task complexity with MDS-UPDRS-III items requiring simultaneous evaluation of multiple movement cues exhibiting observably lower consensus than tasks with discrete actions. By depicting task complexity as the number of behavioral cues a rater must track, we reveal a strong inverse relationship between task complexity and rater consensus. This complexity-consensus relationship emphasizes that variability in clinical scoring is not coincidental but systematic, and constitutes a key element that any automated approach must address. In particular, low consensus indicates that single-rater labels may encode rater-specific biases rather than a stable clinical signal, risking training models that converge towards the tendencies of an individual rater. Our findings motivate a shift in how automated systems should predict clinical ratings, estimating the expected consensus across raters for a given task.

By collecting multiple independent ratings per video, we demonstrate that expert score distributions contain stable, task-dependent structure. We leverage these distributions to predict both mean severity estimates and its associated uncertainty which generalize across visually rated MDS-UPDRS-III tasks. In turn, we reliably distinguish between medication states and show that PARQ predictions for ON medication states generally trend downward as compared to OFF medication states, reflecting the expected clinical improvement. We further evaluated the composite MDS-UPDRS-III score based on the visually rated tasks and demonstrated that PARQ predicted composite scores closely followed the average of multiple raters, even in cases with substantial inter-rater variability. We challenge the assumption that every task is equally hard to rate and thereby requires learning related to the consensus of the task with a distribution-aware deep learning platform. The proposed hybrid learning model in PARQ combining clinical priors with learned features supports robust performance in a sample-efficient manner. By explicitly modeling rating distributions, PARQ adapts to task-dependent variability and reflects expert scoring behavior.

Rather than treating the variability as noise, PARQ directly incorporates it into the learning process to provide a severity estimate that reflects the expected range of expert interpretations. With our dual-branch architecture, we are able to leverage high-dimensional visual information while simultaneously enforcing clinically meaningful structure through task-specific representations. Our hybrid strategy bridges a gap highlighted in recent reviews [24] of using computer vision to analyze movement disorders which is that landmark-only models discard clinically meaningful visual information, whereas end-to-end CNNs offer little interpretability.

In relation to prior work, most computational efforts in PD motor severity assessment have relied on wearable sensors, rule-based heuristics, or models trained on single rater labels. These strategies may adapt poorly across clinical environments and risk embedding individual bias into automated systems. We address these limitations by using data-driven methods and learning from multi-rater annotations. By predicting both a severity estimate and uncertainty measure, PARQ reduces sensitivity to individual scoring preferences. Evaluations using our curated dataset demonstrate that PARQ achieves strong alignment with expert ratings across all tasks. PARQ achieved over 90% accuracy on high task complexity, high-consensus tasks and approximately 80% on low consensus tasks.

The clinical significance of explicitly modeling rating distributions lies in the inherent variability of expert assessments. It aligns with prior observations that rater motor scores embed multiple latent sub-components such as rhythm, amplitude, hesitations, and smoothness, that may not be weighed consistently across experts. In addition to the inherent variability, time constraints and limited expert availability further hinder consistent clinical assessment. Our findings also have implications for the broader use of video-based assessment in clinical care. As telemedicine becomes more widely adopted, there is an eminent need for standardized and reproducible tools that can be deployed beyond specialized centers. With PARQ’s lightweight computational requirements and device agnostic input, we are suitable for integration into routine clinical visits or telemedicine platforms, with the potential to increase access to expert-level assessment for patients in regions with limited availability of movement disorder specialists. Additionally, PARQ could enable dense symptom tracking between episodic in-person evaluations for both clinical care and research.

Several limitations should be acknowledged. The pose estimation libraries were restricted to 2D joint coordinates, which may omit clinically relevant depth information. Future work may incorporate 3D pose estimation using RGB-D sensors or multi-view fusion to enhance signal fidelity without compromising usability. Although landmark reproducibility is a known concern in the literature, we demonstrate the use of PARQ across multiple state-of-the-art pose frameworks (see more in Supplementary Figure 3). PARQ currently focuses on visually assessable items and does not address components such as rigidity, speech, or tremor at rest in all contexts; extending to these domains will require multi-modal input like audio and wearable sensors. While the current dataset was collected in a single academic center, ongoing efforts will expand the platform to multi-site cohorts. Additionally, while PARQ was predictive for the three expert raters for our study, we would need a larger sample of raters to ensure its utility. To compare alternative approaches by different investigators, access to common databases is required with strict privacy preserving measures.

Future work will aim to expand the range of supported tasks and evaluate the system in more diverse clinical settings. Federated or privacy-preserving learning may help address rater of site-specific biases. Further investigation is also necessary to see how uncertainty estimates can inform clinical decision-making. By modeling both severity and uncertainty in a clinically interpretable manner, PARQ provides a platform for more standardized and data-driven assessment of motor symptoms.

## Supplementary Material

Supplementary Files

This is a list of supplementary files associated with this preprint. Click to download.

- PARQSupplementaryIshaChakraborty0130.pdf

## Figures and Tables

**Table 1:** PARQ Predictions across MDS-UPDRS-III tasks Values report accuracy of predictions of samples within ±1 class of the average of the ratings, within 1 standard deviation, and within 1.5 standard deviations for each MDS-UPDRS-III task.

Task	Within ±1	Within 1 std dev	Within 1.5 std dev
Finger Tapping	0.89	0.43	0.63
Hand Movement	0.95	0.58	0.80
Pronation–Supination	0.79	0.48	0.77
Toe Tapping	0.90	0.60	0.78
Leg Agility	0.88	0.38	0.60
Arising from Chair	0.93	0.68	0.68
Gait	0.86	0.48	0.59
Postural Stability	0.74	0.39	0.52

**Table 2:** PARQ predictions stratified by medication state (within ±1 of average ratings). Values report accuracy of predictions of samples within ±1 class of the average of the ratings combined and separately for OFF and ON medication states

Task	All (Within ±1)	Off Meds (Within ±1)	On Meds (Within ±1)
Finger Tapping	0.89	0.90	0.85
Hand Movement	0.95	0.99	0.85
Pronation–Supination	0.79	0.90	0.71
Toe Tapping	0.95	0.95	0.95
Leg Agility	0.88	0.85	0.80
Arising from Chair	0.93	0.99	0.90
Gait	0.86	0.90	0.85
Postural Stability	0.74	0.78	0.72

**Table 3:** MDS-UPDRS-III Task-specific Signal Computation

Task	Signal Equation	Description
**3.4 Finger Tapping**	$d(t)={\left\|p_{\text{thumb}}(t)-p_{\text{index}}(t)\right\|}_{2}$	Thumb-index distance quantifies tap cycles.
**3.5 Hand Movement**	$d_{\text{hand}}(t)=\frac{1}{n}\sum_{i=1}^{n}{\left\|p_{i}(t)-p_{\text{palm}}(t)\right\|}_{2}$	Mean fingertip-to-palm distance tracks hand opening and closing.
**3.6 Pronation-Supination**	$\Delta y_{\text{wrist}}(t)=y_{\text{wrist}}(t)-y_{\text{wrist}}\left(t_{0}\right)$	Vertical wrist displacement captures supination-pronation flips.
**3.7 Toe Tapping**	$\Delta y_{\text{toe}}(t)=y_{\text{toe}}(t)-y_{\text{toe}}\left(t_{0}\right)$	Toe vertical movement tracks tap amplitude
**3.8 Leg Agility**	$\Delta y_{\text{knee}}(t)=y_{\text{knee}}(t)-y_{\text{knee}}\left(t_{0}\right)$	Knee vertical displacement during rapid leg lifts.
**3.9 Arising from Chair**	$\Delta y_{\text{torso}}(t)=y_{\text{torso}}(t)-y_{\text{torso}}\left(t_{0}\right)$	Torso vertical rise measures standing effort.
**3.10 Gait**	$\Delta d_{\text{step}}\left(t_{s}\right)=d_{\text{step}}\left(t_{s}\right)-d_{\text{step}}\left(t_{0}\right)$	Step length derived from inter-ankle distance during walking.
**3.12 Postural Stability**	$\Delta y_{\text{torso}}(t)=p_{\text{torso}}^{y}(t)-p_{\text{torso}}^{y}\left(t_{\text{pull}}\right)$	Recovery displacement of the torso following backward perturbation.

**Table 4:** Summary of the common Task-Specific Kinematic Features used across all tasks. Each feature is computed on the subset of joints relevant for each task.

Feature	Description
Avg Vel	Average velocity across the movement signal
Avg Acc	Average acceleration (rate of change of velocity)
Avg Jerk	Average jerk (rate of change of acceleration)
Avg Freq	Mean dominant frequency of the signal
Max Fft	Maximum spectral power from FFT
Avg Peak Dist	Mean distance between successive peaks
Avg Trough Dist	Mean distance between successive troughs
Avg Amp	Mean peak-to-trough amplitude
Amp Std	Standard deviation of amplitude over time
Dec Amp	Linear decay in amplitude over the task duration
Dec Vel	Linear decay in velocity over the task duration
Period Range	Range of time between consecutive movement cycles

## Data Availability

The full dataset used in this work cannot be publicly shared due to patient privacy and confidentiality. Upon reasonable request, de-identified summary data can be made available from the corresponding author.

## Code Availability

The code for this study is not publicly available but may be made available to qualified researchers on reasonable request from the corresponding author.
